# Beyond effectiveness in eHealth trials: Process evaluation of a stepped-care programme to support healthcare workers with psychological distress (RESPOND-HCWs)

**DOI:** 10.1177/20552076241287678

**Published:** 2024-10-18

**Authors:** Roberto Mediavilla, Blanca García-Vázquez, Kerry R. McGreevy, James Underhill, Carmen Bayón, María-Fe Bravo-Ortiz, Ainoa Muñoz-Sanjosé, Josep Maria Haro, Anna Monistrol-Mula, Pablo Nicaise, Papoula Petri-Romão, David McDaid, A-La Park, Maria Melchior, Cécile Vuillermoz, Giulia Turrini, Beatrice Compri, Marianna Purgato, Rinske Roos, Anke B. Witteveen, Marit Sijbrandij, Richard A. Bryant, Daniela Fuhr, José Luis Ayuso-Mateos

**Affiliations:** 1 Department of Psychiatry, 16722 Universidad Autónoma de Madrid (UAM), Madrid, Spain; 2 Instituto de Investigación Sanitaria del 16517 Hospital Universitario La Princesa, Madrid, Spain; 3 456032 Centro de Investigación Biomédica en Red de Salud Mental (CIBERSAM), Instituto de Salud Carlos III, Madrid, Spain; 4 Department of Psychiatry, Clinical Psychology, and Mental Health, 16268 Hospital Universitario La Paz, Madrid, Spain; 5 638528 Instituto de Investigación Sanitaria del Hospital Universitario La Paz (IdiPAZ), Madrid, Spain; 6 Research Consultant, Brighton, UK; 7 221703 Parc Sanitari Sant Joan de Deu, IRSJD, Sant Boi de Llobregat, Barcelona, Spain; 8 Institute of Health and Society, 83415 UCLouvain, Belgium; 9 539160 Leibniz Institute for Resilience Research, Mainz, Germany; 10 Care Policy and Evaluation Centre, Department of Health Policy, 4905 London School of Economics and Political Science, London, UK; 11 27063 Sorbonne Université, INSERM, Institut Pierre Louis d’Épidémiologie Et de Santé Publique, IPLESP, Equipe de Recherche en Epidémiologie Sociale, ERES, Paris, France; 12 WHO Collaborating Centre for Research and Training in Mental Health and Service Evaluation, Department of Neuroscience, Biomedicine and Movement Science, Section of Psychiatry, 19051 University of Verona, Verona, Italy; 13 Department of Clinical, Neuro- and Developmental Psychology, WHO Collaborating Center for Research and Dissemination of Psychological Interventions, Amsterdam Public Health Research Institute, 1190 Vrije Universiteit, Amsterdam, The Netherlands; 14 School of Psychology, 441985 University of New South Wales, Sydney, Australia; 15 39967 Leibniz Institute for Prevention Research and Epidemiology – BIPS, Bremen, Germany; 16 Health Sciences, 9168 University of Bremen, Bremen, Germany; 17 Department of Health Services Research and Policy, (4906 London School of Hygiene and Tropical Medicine, London, UK

**Keywords:** Mental health, healthcare workers, process assessment, health care, eHealth, internet-based intervention, qualitative evaluation, quantitative evaluation

## Abstract

**Objectives:**

This study presents the process evaluation of an effective stepped-care programme of eHealth interventions (Doing What Matters in Times of Stress [DWM] and Problem Management Plus [PM+]) for healthcare workers (HCWs) with psychological distress (RESPOND-HCWs trial) conducted in Spain. The aim is to analyse the context in which the programme was delivered, assess key implementation outcomes and explore mechanisms of action.

**Methods:**

We used mixed methods. Quantitative data came from routine randomised control trial monitoring and structured observation, and qualitative data were collected using semi-structured, in-depth interviews with trial participants (*n* = 12) and decision-makers (*n* = 7) and a focus group discussion with intervention providers (*n* = 7). We conducted a descriptive analysis of quantitative data using R software and a thematic analysis of qualitative data using NVivo.

**Results:**

Context analysis revealed implementation barriers, including unrealistic expectations of participants about the programme and mental health-related stigma. The flexibility of interventions and the opportunity for mental health actions were enabling factors. Implementation outcomes showed that the trial was feasible, appropriate and timely, and that the intervention was delivered with minimal protocol deviations and good acceptance among participants. Mechanisms of action included confidence in the positive effect of the intervention, a good therapeutic relationship and specific intervention components.

**Conclusions:**

These results supplement the outcome evaluation and can help inform large-scale implementation in similar settings. Specific recommendations include increasing mental health awareness and reducing stigma in the implementation setting, including a short orientation session and ensuring flexibility in schedules and peer support.

**Trial registration number:**

NCT04980326.

## Introduction

Public health and policy require a comprehensive understanding of which health strategies are effective and in which contexts. In the last two decades, public health research has been increasingly informing decision-making using evidence from three areas: the causes and risk factors of diseases, the relative impact of interventions and the context in which such interventions can be successfully implemented.^1^ High-quality studies, such as nationwide cohorts or meta-analyses of randomised controlled trials (RCTs), provide strong results in the first two areas. However, evidence-based public health still needs to generate more evidence regarding the implementation of particular interventions or strategies in specific contexts and under real-life conditions.^2,3^ This lack of implementation evidence hampers the translation from evidence-based interventions to routine practice.^4^

The COVID-19 pandemic posed additional challenges to the implementation of interventions, including evidence-based mental health programmes and strategies. In 2020, only one in three countries reported having systems in place for mental health and psychosocial preparedness for emergencies and/or disasters such as pandemics.^5^ By late 2021, an umbrella review identified that the implementation of remote delivery mental health support faced barriers, affecting both practitioners (e.g., concerns about the therapeutic alliance during online delivery of interventions, exhaustion) and organisations (e.g., insufficient technical support, lack of funding).^6^ Of note, these barriers can contribute to longer waiting times and an increased risk of symptoms becoming chronic,^7^ resulting in preventable suffering and high economic costs.^8^

Spain was one of the earliest COVID-19 pandemic hotspots worldwide. As of May 2020, point prevalence estimates of SARS-CoV-2 infections in the metropolitan areas of Madrid and Barcelona were 11.3% and 7%, respectively,^9,10^ and one in four confirmed cases were healthcare workers (HCWs).^11^ Amidst this crisis, the European Commission funded the project ‘Preparedness of health systems to reduce mental health and psychosocial concerns resulting from the COVID-19 pandemic’ (RESPOND). The study involved four RCTs across various vulnerable populations experiencing high levels of psychological distress, including HCWs employed by the Departments of Health of the Community of Madrid and in Catalonia,^12^ as well as Polish migrant workers living in the Netherlands,^13^ migrant populations resettled in Italy^14^ and people without stable housing conditions in France.^15^ The overall goal was to provide decision-makers with scalable and effective psychological interventions during epidemics and other public health emergencies.

The Spanish trial (RESPOND-HCWs) involved three phases that took place between December 2020 and September 2022. During phase 1 (December 2020–May 2021), we used qualitative data from 97 informants to locally adapt two World Health Organization (WHO) interventions into a remotely delivered, stepped-care programme.^16^ The first intervention consisted of a stress management course based on the Self Help Plus (SH+) booklet called *Doing What Matters in Times of Stress* (DWM).^17^ It was adapted into a guided self-help web application consisting of five modules that included strategies based on acceptance and commitment therapy, mindfulness techniques and audio recordings that further explained and reinforced the use of the techniques. The course was delivered in 6 weeks and included 15-minute weekly support calls. The second step was a five-session transdiagnostic intervention called Problem Management Plus (PM+) that included cognitive-behavioural and problem-solving techniques.^18^ It involved five individual 1-hour weekly sessions that were delivered through videoconference. During phase 2 (November 2021–August 2022), we examined the effectiveness of the stepped-care programme in an RCT involving HCWs experiencing self-reported psychological distress. Intervention providers were trainees in psychiatry, clinical psychology and mental health nursing, closely supervised by senior mental health practitioners. The interventions, including the DWM app, were delivered in Spanish and/or Catalan. We found moderate-to-large reductions in anxiety, depression, and posttraumatic stress disorder symptoms in the intervention arm, compared to enhanced care as usual at 5 months after baseline assessment.^19^ This outcome evaluation provided decision-makers with an effective open-access stepped-care programme adapted to crisis settings where evidence-based interventions were scarce, such as health care settings during the COVID-19 pandemic.^20,21^ Finally, during phase 3 (June 2022–September 2022), we collected qualitative data from key stakeholders to further analyse the trial implementation.

In this study, we present the results of the process evaluation of the RESPOND-HCWs study, which uses data from all three trial phases. In contrast to outcome evaluations, which rarely guide the translation of trial findings into feasible practice, process evaluations answer broader research questions and can rapidly inform decision-making.^22,23^ Accordingly, we used mixed methods and multiple data sources to (a) describe the context in which the stepped-care programme was delivered, (b) analyse key implementation outcomes, and (c) explore the mechanisms of action through which the programme improved mental health. We followed the United Kingdom's Medical Research Council's (MRC) guidelines to structure the process evaluation,^22,23^ the implementation research model by Proctor and colleagues to choose implementation outcomes^24,25^ and guidelines for conducting thematic analysis in psychology to analyse qualitative data.^26^

## Methods

### Data collection and data sources

We used mixed methods to analyse data from multiple sources collected during the RESPOND-HCWs trial, as specified in the study protocol.^12^ We collected qualitative and quantitative data across the three phases of the trial, and we did not reimburse any informant for their participation in the evaluations. During phase 3, we conducted in-depth interviews with a purposive sample of trial participants (*n* = 12) and decision-makers (*n* = 7) and a focus group discussion with intervention providers (*n* = 7). In-depth interviews were done remotely (phone or Microsoft Teams) and the focus group discussion was conducted in person at the School of Medicine, Universidad Autónoma de Madrid. All participants provided their written informed consent to participate in this study. Table 1 shows the characteristics of participants recruited in phase 3 (Supplementary Tables 1 and 2, show the characteristics of participants recruited in phases 1 and 2, respectively). The English versions of the interview scripts, developed ad hoc, are provided in the Supplementary File 1: Interview and Focus Group Scripts.

**Table 1.** Characteristics of informants recruited in phase 3.

**Informant**	**Gender**	**Age**	**Position**
*Decision-makers*			
DM1	Male	30–44	Primary care centre – Management
DM2	Female	45–60	Major public hospital – Continuing Care Department
DM3	Male	30–44	Madrilenian Department of Health – Support programme for HCWs
DM4	Female	45–60	Major public hospital – Nursing Department
DM5	Male	45–60	Madrilenian Department of Health – Primary Care Division
DM6	Female	45–60	Madrilenian Department of Health – Mental Health Division
DM7	Female	45–60	Major public hospital – Emergency Department
*Trial participants*			
TP1	Female	45–60	Nurse
TP2	Female	30–44	Nurse
TP3	Male	<30	Resident
TP4	Female	45–60	Nurse
TP5	Female	30–44	Nurse
TP6	Male	30–44	Nursing technician
TP7	Female	<30	Nurse
TP8	Female	45–60	General practitioner
TP9	Female	45–60	Nursing technician
TP10	Female	<30	Nursing technician
TP11	Female	45–60	General practitioner
TP12	Female	45–60	Nursing technician
*Intervention providers*			
IP1	Male	<30	Resident (first year), psychiatry
IP2	Female	<30	Resident (first year), mental health nursing
IP3	Female	<30	Resident (first year), mental health nursing
IP4	Male	<30	Resident (first year), psychiatry
IP5	Female	<30	Resident (first year), psychiatry
IP6	Male	<30	Resident (first year), mental health nursing

*Note*. DM = decision-maker; HCWs = health care workers; TP = trail participants; IP = intervention providers.

**Figure 1.**
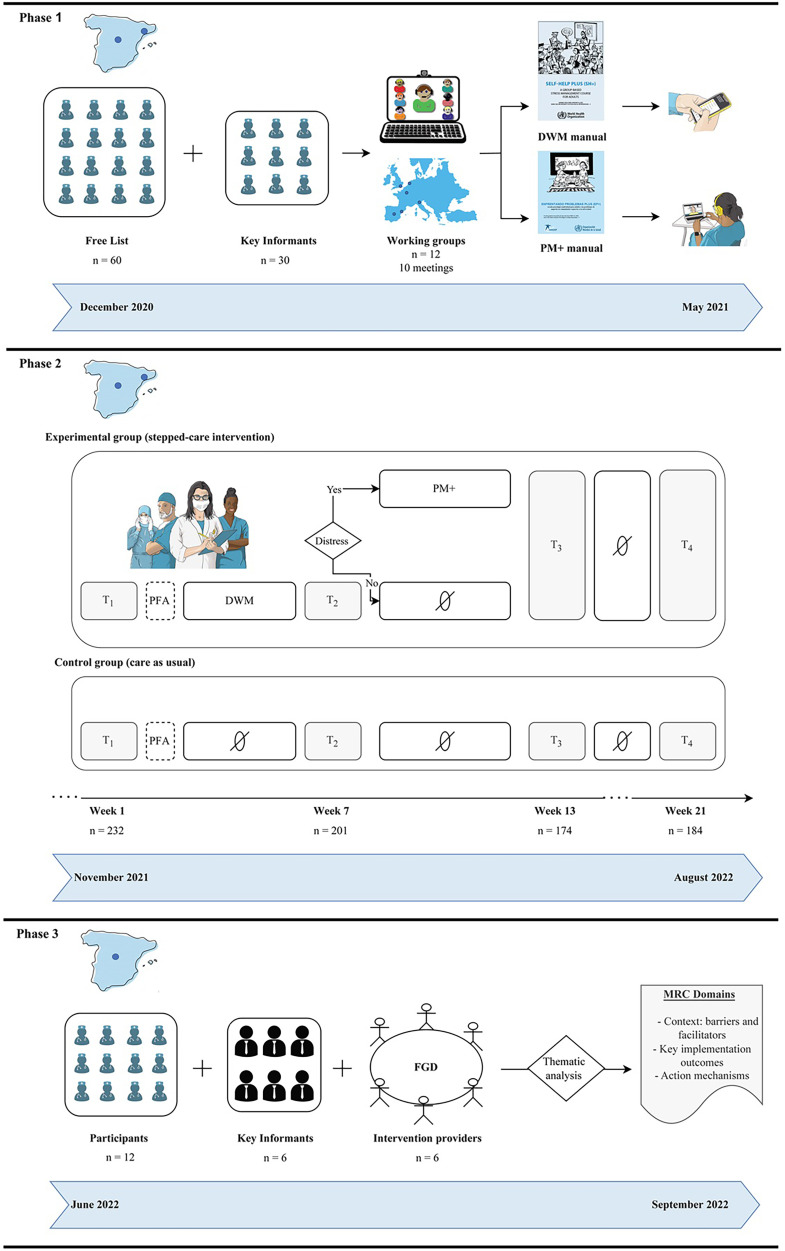
Overview of the trial phases.

The RCT took place in the Community of Madrid and in Catalonia, but its phase 3 data were available only in the former. All data were collected prior to the analysis of the trial outcomes, which means that neither the researchers nor the participants knew the intervention was effective – we only had access to key RCT process data, such as dropout rates or the proportion of adverse events reported. An overview of the phases of the trial is shown in Figure 1.

We structured the data collection and analysis following the UK MRC's framework for process evaluations of complex interventions,^22,23^ which includes three main components: the context in which the stepped-care programme was delivered, the assessment of key implementation outcomes, and the mechanisms of action through which the interventions may work. Table 2 summarises data collection methods and sources across trial phases that were used to inform the process evaluation components.

**Table 2.** Data collection methods, sources, and phases, used across MRC framework's themes.

MRC: Component	MRC: Methods	Source	Phase	Description
*Context*				
Contextual factors that affect (and may be affected by) implementation, mechanisms and outcomes	Stakeholder interviews	Decision makers, mental health experts and potential end-users of interventions	#1. Local adaptation	DWM and PM+ local adaptations were undertaken by a working group led by WHO. Main feedback came from in-depth interviews with 96 stakeholders at both study sites and end-user testing
	Participant interviews	Trial participants (subsample, intervention arm)	#3. Process evaluation	Participants assigned to the intervention arm reported the most common barriers and enabling factors that they experienced with DWM and PM+ (*n* = 12, individual interviews)
	Stakeholder interviews	Decision makers	#3. Process evaluation	Decision-makers reported barriers and enabling factors to scalability within the Madrilenian Department of Health (n = 7, individual interviews)
	Implementer interviews (focus group discussions)	Intervention providers (peers)	#3. Process evaluation	Intervention providers described what they found most challenging when delivering the programme and suggested how to overcome such challenges (n = 6, focus group discussion)
*Implementation*				
Feasibility	Administrative data	Routine monitoring data	#2. RCT	Number of participants who reached out to take part in the trial; recruitment and retention rates; primary endpoint collection rate; recruitment and retention rates (intervention providers); attendance to supervision sessions; delays in follow-up assessments
Appropriateness/usefulness	Stakeholder interviews	Decision makers	#3. Process evaluation	Decision-makers were asked whether the stepped-care programme was appropriate and timely
Fidelity	Structured observations	Supervisors	#2. RCT	Supervisors revised a random sample of DWM calls and PM+ videocalls and scored them based on criteria developed by intervention providers
	Implementer self-report	Routine monitoring data	#2. RCT	Deviations from expected duration of DWM and PM+ calls and videocalls
Acceptability/satisfaction	Administrative data	Routine monitoring data	#2. RCT	Attrition rates (incl. non-use attrition, as defined by the proportion of participants not accessing DWM website and not attending any PM+ sessions [if applicable])
	Participant interviews	Trial participants (subsample, intervention arm)	#3. Process evaluation	Intervention users reflected upon acceptability of the stepped-care format, the remote delivery and the peer support
*Mechanisms of action*				
Participants’ responses to and interactions with the intervention	Participant interviews	Trial participants (subsample, intervention arm)	#3. Process evaluation	Participants said whether the intervention had worked for them -and, if so, why they thought it worked
Unexpected pathways and consequences	Administrative data	Routine monitoring data	#2. RCT	Serious adverse events and incidental findings were reported to the Ethics Committee

*Note*. DWM = doing what matters in times of stress; PM+ = problem management plus; WHO = World Health Organization; RCT = randomized controlled trial.

#### Context

In the first phase of the RCT (phase 1), we conducted in-depth interviews with local stakeholders (frontline HCWs, mental health practitioners and decision-makers) to understand the context and the mental and occupational health strategies in place. We identified several occupational and mental health problems that frontline HCWs faced one year after the initial COVID-19 outbreak and some unmet needs. The results are available elsewhere^16^ and were used to adapt DWM and PM+ and to tailor them to HCWs working in Spain amidst the COVID-19 pandemic. At trial completion (phase 3), we conducted (a) in-depth interviews with participants allocated to the intervention arm and with decision-makers (policymakers and/or mental health experts) and (b) a focus group discussion with DWM and PM+ providers. We used them to collect data for three MRC themes (context, implementation outcomes, and mechanisms of action). Regarding context, we asked informants about the barriers and enabling factors for intervention implementation in the Madrilenian Department of Health based on their experience, as either users and providers or decision-makers.

#### Implementation outcomes

Implementation outcomes were measured during and after the trial – phases 2 and 3, respectively. During phase 2, we collected indicators of feasibility (e.g., recruitment rates, availability of primary outcome measure), fidelity (adherence to DWM and PM+ intervention protocols and quality of delivery) and acceptability (attrition rates, preferences for text versus phone weekly support contacts in DWM). Methods included structured observation of audio and video recordings of the sessions, implementer self-reports and administrative data. Audio and video recordings covered approximately 10% of all contacts, with DWM first and last calls being deliberately overrepresented. Supervisors listened to DWM records or watched PM+ videos and rated them using a structured checklist developed by WHO (see Supplementary File 2: Supervision Checklist). During phase 3, we used interviews with decision-makers to analyse appropriateness, as well as with trial participants, to analyse acceptability. All implementation outcomes were described and analysed following Proctor’s guidelines^24^ (see Table 3).

#### Mechanisms of action

During phase 3, we asked trial participants how they thought the intervention had worked for them. We used this information to identify mechanisms that help understand the positive effects of the intervention and to inform potential future adaptations of the programme.

### Procedures and data analysis

Quantitative data came from routine monitoring during the RCT and structured observation checklists used by senior supervisors. We used descriptive statistics such as means and standard deviations, medians and interquartile ranges, or frequencies and percentages to describe the results. Analyses were carried out using R (version 4.3.0) and R Studio.

**Table 3.** Description of implementation outcomes .

Outcome	Definition
Feasibility	Feasibility is defined as the extent to which a new treatment, or an innovation, can be successfully used or carried out within a given agency or setting
Appropriateness/usefulness	Appropriateness is the perceived fit, relevance, or compatibility of the innovation or evidence-based practice for a given practice setting, provider, or consumer; and/or the perceived fit of the innovation to address a particular issue or problem
Fidelity/adherence	Fidelity is defined as the degree to which an intervention was implemented as it was prescribed in the original protocol or as it was intended by the program developers
Acceptability/satisfaction	Acceptability is the perception among implementation stakeholders that a given treatment, service, practice, or innovation is agreeable, palatable, or satisfactory

*Note*. Adapted from Proctor et al. (2011).

Qualitative data were collected using in-depth interviews and focus group discussions at trial completion. In-depth interviews were performed by two authors: RM (Ph.D., psychologist, male) and BG-V (Ph.D. candidate, psychologist, female). The researchers did not have any relationship with the participants before the study started. The participants were informed about the objectives of the study both during the interviews and in the information sheet. Recordings were transcribed using an automated transcription assistant for audio data (NVivo 11 – Transcription). One person (BG-V) coded the transcripts to formulate themes following Braun and Clarke's guidelines for thematic analysis^26^ and using the MRC's framework components (context, implementation, and mechanisms of action) as the coding frame. Another person (RM) independently coded the first two interviews to discuss discrepancies. Our approach to data analysis was essentialist/realist, and the level of content was explicit (i.e., semantic). Next, the most representative quotes were extracted in a text document and used to summarise the main results of each component. The focus group discussion was conducted by one of the authors (RM). We asked intervention providers to name the challenges (i.e., implementation barriers) experienced during the implementation of DWM and PM+. Following a short discussion, we asked them to cluster, label and order the challenges, from most to least frequent, using coloured paper sheets. Last, we asked them to think of ways of overcoming the implementation barriers. Another author (BG-V) summarised the main barriers and their potential resolution. The COREQ (COnsolidated criteria for REporting Qualitative research) checklist is provided as a supplementary file.

Pseudonymised data supporting quantitative analysis and qualitative data (audio recordings and their transcription) are not available in compliance with the European General Data Protection Regulation. Anonymised data are available from the corresponding author upon request.

## Results

### Context

Before the RCT started (phase 1), the RESPOND consortium created a working group with representatives from all RESPOND coordinating sites and the WHO to adapt and tailor DWM and PM+ to the target populations locally. This group met 10 times over a 6-month period and analysed interviews with key stakeholders from each trial location. The working group used these inputs to produce a final list of contextual adaptations, which essentially included format changes (e.g., DWM delivered over the phone and a website, PM+ support via videoconference) and content (e.g., scenes involving HCWs fighting the COVID-19 pandemic). The adaptations of DWM and PM+ are shown in Table 4 and the sessions’ content is shown in Supplementary Figure 1: DWM and PM+ Components.

**Table 4.** DWM and PM+ adaptations*.*

DWM	PM+
In the RESPOND study, pre and post intervention assessments will be carried out by trained researchers. Sections on pre and post assessments have been removed from the manual.	In the RESPOND study, PM+ will be delivered remotely through teleconferencing. All helpers should complete the EQUIP ‘Delivering Remote Services’ training which can be accessed here: https://whoequipremote.org/en-gb?category = all. A summary of guidelines for remote delivery of PM+ have been included in the ‘Setting’ section
Screen shots for the DWM online platform developed specifically for the RESPOND project have been included to illustrate how the intervention will look for participants and provide instruction for helpers on navigating the ‘coach’ pages of the platform.	Specific considerations to address concerns about confidentiality that may arise with this specific participant group are included.
Originally this manual was designed for face-to-face support or live support over the phone. Due to the different needs of the RESPOND study participant groups, guidance and templates for sending asynchronous messages have been included and a standardised protocol for contacting and sending out messages across sites developed.	A guideline for encouraging discussion about the impact of COVID-19 in the ‘understanding adversity’ section has been included. This includes additional sentences for the script to explain how worry and COVID-19 are linked, and other concerns/stressors specific to RESPOND study target populations as identified during the qualitative research phase.
A section on supporting participants during the COVID-19 pandemic has been included.	Suggested adaptations including changes to the script for COVID-19 are included in the managing stress, managing problems, get going keep doing and social support sections
Throughout the manual, the word ‘participant’ was used instead of ‘client’.	Suggestions are included in the managing stress and managing problems sections for integrating techniques from DWM. These are recommendations for participants who found techniques from DWM helpful. At the beginning of PM+ helpers should ask participants if they found any of the techniques from DWM and should refer to training materials when conducting this discussion. Helpers should follow the standard PM+ protocol if the participant did not find DWM useful or failed to practice any of the techniques. Helpers should keep a copy of the DWM book, ask participants if they found DWM helpful and if so, which techniques they used/found helpful grounding, unhooking, being kind, acting on your values, making room).
New images were included to show common problems for target populations amidst the COVID-19 pandemic	Case examples have been adapted throughout the manual to be relevant to the study populations and the difficulties they face.
	Yellow boxes have been added at the beginning of relevant chapters to highlight changes and encourage helpers to consider specific lessons relevant to the RESPOND adaptation during the training.

*Note* DWM = doing what matters in times of stress; PM = problem management plus; RESPOND = Preparedness of health systems to reduce mental health and psychosocial concerns resulting from the COVID-19 pandemic.

At trial completion (phase 3), we identified implementation barriers and enabling factors using in-depth interviews with trial participants and decision-makers (see Table 5).

**Table 5.** Implementation barriers and enabling factors reported by trial participants and decision-makers

Name	Description	Quote	Frequency
*Implementation barriers*			
DWM visual design	DWM app design was inherited from the original DWM booklet, and some participants did not like it	*As I told your colleague, I was not too fond of the artwork. I thought, gosh, only dummies, how grim!* (TP8)	2 TP
Confidentiality	Intervention providers and trial participants were colleagues and could cross into each other in the workplace	*Thinking about it, being from the same professional association. I did not like it at all, but it was the only possibility.* (TP2)	2 TP, 1 DM
Practice	Intervention providers asked trial participants to do DWM and PM+ exercises between sessions	*Yes, health workers usually see it as another requirement to be better off; what you should do to take better care of yourself. Then, you must do this in the app, reserve a while, and learn.* (DM3)	3 TP, 3 DM
Stigma	Stigma towards mental health problems may reduce help-seeking behaviours	*No, because they might feel their problem isn’t serious enough to respond to treatment, they downplay it or think they can solve it on their own. (TP7)*	4 TP, 2 DM
Unrealistic expectations about the stepped-care programme	The step care consisted of a guided self-help stress reduction course (DWM) and a 5-session CBT-based programme which could be labelled as ‘low-intensity’ psychological interventions	*I understand some people saying they don’t want an application and, in a way, say it is not enough.* (DM1). *If you don’t feel well, you want more intensive, personalised care. But that is, of course, not possible. The programme is very wide. I suppose it is designed this way to help with things that are not very specific.* (TP8)	5 TP, 4 DM
DWM phone calls	DWM was supported by six weekly 15-min phone calls –no other contacts were made between intervention providers and trial participants	*The calls were made by phone so that you couldn't see the person, and I didn't even know them. I didn’t feel like talking on the phone much.* (TP2)	4 TP, 1 DM
Trust in the Madrilenian Health System	HCWs may not want to take part in this or similar programmes if they come from health office managers	*Yes, I think so. People are not going to be open to anything that the administration proposes.* (TP7)	1 TP, 2 DM
Internet literacy	DWM was delivered via web app, and PM+ was delivered via Teams video calls	*You might lose the closest contact with the other person* (TP11). *A substantial number of workers are around 50 and 60 years old and might have a barrier due to less familiarity with technology.* (DM3)	7 TP, 3 DM
Intervention provider profile	Intervention providers were psychiatry, clinical psychology and mental health nursing trainees	*I am not sure about the nurses. For example, when it is a psychiatrist or psychologist, we are talking about psychological support; when it's a nurse who does it, I’m not sure*. (DM2)	2 DM
*Enabling factors*			
Flexible schedules	DWM and PM+ calls were scheduled according to the preferences of the participant	*Usually, they would confirm the same day or the day before, and I would have their email just in case I couldn’t attend. Although, to be honest, I was always available; if I had any problems, I could notify them in advance.* (TP2)	6 TP, 1 DM
Remote delivery	DWM and PM+ were both delivered fully remote	*Moreover, for example, they can do it on their own at home whenever they want, and it is online, or they have the possibility to do it at work.* (DM5)	1 TP, 6 DM
Increasing sensitivity towards mental health problems	The Spanish social and political context is becoming more sensitive towards mental health –this includes HCWs’ mental health, which was heavily burdened during the COVID-19 pandemic	*I think the Regional Mental Health Office of Madrid. It's working hard for the population's mental health needs. That mental health problems have increased a lot is a reality, or they were present before, but they were not known as much.* (DM6)	5 DM

*Note*. DM = decision maker; DWM = doing what matters in times of stress; HCW = health care worker; PM+ = problem management plus; TP = trial participant.

Participants frequently mentioned that potential users could see the stepped-care programme as too low in intensity, especially DWM, and this was somewhat related to the remote delivery. Decision-makers were also concerned about the intensity of the programme and about the delivery format, especially if it brings inequality based on the level of Internet literacy. Interestingly, both trial participants and decision-makers outlined the contribution of a key enabling factor, namely flexible schedules and remote delivery, to the implementation and potential effectiveness of the stepped-care programme. One participant said that she could change or modify the appointment even on the day. Another decision-maker said that participants could practice either at home or at work, based on their preferences. Mental-health-related stigma was another key barrier, mainly internalised stigma. For instance, one participant mentioned that people downplay their mental well-being and ignore or minimise their symptoms. Even though some decision-makers also highlighted the harmful effects of mental health-related stigma, they also mentioned that the COVID-19 pandemic raised mental health awareness and created opportunities for mental health actions within the Madrilenian Department of Health.

Intervention providers also identified barriers separately for DWM (step 1) and PM+ (step 2). For DWM, they highlighted the lack of flexibility of the duration of phone calls (supervisors were quite strict considering the RCT protocol), difficulties in finding a suitable schedule both for intervention providers and participants, relative lack of feedback during guided practices on the mobile phone, technical problems with the app, inaccurate expectations about a low-intensity self-help course such as DWM and high levels of psychological distress among many participants. For PM+, intervention providers reported barriers similar to those of DWM (flexibility, technical problems and time pressure), in addition to some specific to PM+, namely its highly structured protocol (perceived by both intervention providers and participants as somehow constraining) and the resistance of some participants to talk about their problems when they have to work with real examples. They mentioned two ways to solve technical issues: making DWM and PM+ interfaces more usable and friendly and providing intervention providers with specific training to help users overcome technical problems. They also suggested that intervention protocols, especially PM+, could be more flexible and build on more general objectives for each session – even though they were aware of the restrictions and the need for high control in RCTs.

Last, we asked decision-makers how this strategy could successfully be implemented in the Madrilenian Department of Health. They said that effective scalability would require a general orientation towards mental health care within the Department of Health, as described by one participant: ‘I think [scaling up] would require moving from individual towards more collective attitudes’ (DM1 [decision-maker 1]). They also suggested specific strategies, which included offering support in combination with anti-stigma campaigns: ‘Many people still see [asking for help] as a symptom of weakness. This would help people signing up [for the step care]’ (DM2). Other suggested strategies included coordinating with existing mental health resources (‘This tool should liaise with the mental health support programmes for HCWs in primary care settings’ [DM5]), and keeping the stepped-care format (‘I think it is good that they offer only the first [DWM] or the second [PM+] step of the intervention, depending on your needs (…) because resources are limited’ [DM7]). It was also noted that ideally offering sessions within working hours would be helpful (‘physicians [in primary care centres] could do the sessions or the daily practice between 2 pm and 3 pm [these are still working hours but not with patients]’ [DM1]). When we asked decision-makers if there were any specific units or workers who could especially benefit from this, they said that ancillary workers could be particularly vulnerable:

I mean that usually, the positions with less professional recognition, such as hospital porters or nursing assistants, etc., are exposed to more adverse circumstances and are prone to a mental health history, and, therefore, the episodes are usually more severe and probably need more help. (DM3)

They suggested focusing on HCWs in COVID-related units, emergency departments and primary care settings.

### Implementation outcomes

#### Feasibility

In the Community of Madrid, 207 potential trial participants contacted the study team over a 4-month recruitment period. In all, 170 (82%) were screened for eligibility and 110 (65%) were randomised. The primary endpoint was achieved in eight months, with a response rate of 80%. The median deviation from planned assessment dates in the intervention arm was minimal after DWM and acceptable (1 week) after PM+, showing that the delivery of the interventions was not significantly delayed (see Table 6).

We invited all psychiatry, clinical psychology and mental health nursing trainees at the coordinating centre (Hospital Universitario La Paz, Madrid, Spain) to participate as intervention providers in the trial (*n* = 14). In all, 11 of them accepted, finished the training in 10 weeks, and received 26 supervision sessions over the seven months the trial was ongoing. In addition to intervention providers, we recruited four trainers who effectively delivered both DWM and PM+ training and supervision and completed the quality checks after structured observation of recorded sessions.

**Table 6.** Time between assessments.

Assessments	Control Arm			Intervention Arm		
	Planned Day	Actual Day (median)	Days Delayed	Planned Day	Actual Day (median)	Days Delayed
Post DWM	42	44	2	42	44	2
Post PM+	84	86	2	84	92.5	8.5
Follow-up	140	142.5	2.5	140	142	2

*Note*. DWM = doing what matters in times of stress; PM+ = problem management plus.

#### Appropriateness

Overall, decision-makers thought the intervention was appropriate and timely for the Madrilenian Department of Health. One decision-maker outlined the importance of caring for those who care:

Taking the initiative in the care of the worker indeed seems absolutely right; in other words, you must take care of the carer. If not, we are doing it wrong—the same stands for the patients’ carers. We have to identify who the primary carers are and support them because if we don’t, they will end up giving up. I think it's an interesting initiative. (DM2)

Another stakeholder specifically mentioned that it was timely, even two years after the initial COVID-19 pandemic outbreak: ‘Yes, I like it. I think it's positive and helps because all these people need someone to help and unload them in those moments of stress and overwhelming’ (DM5).

Last, one trial participant (TP) mentioned how this is relevant from a societal perspective: ‘Yes, I think it would be great for everyone, for a lot of people. Yes, because it seems we are an unhappy society; we are not at ease with anything, and I think we could use this’ (TP6).

#### Fidelity

**Figure 2.**
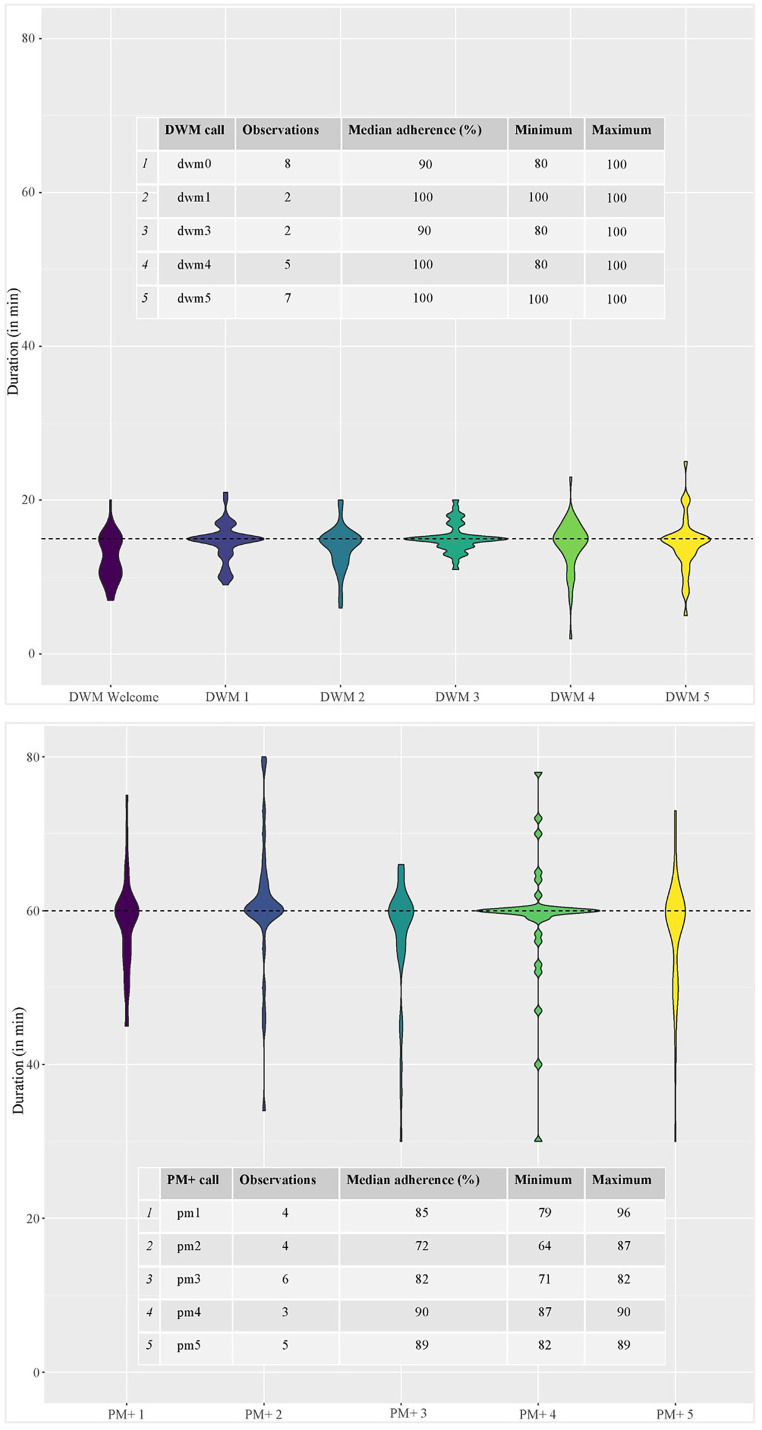
DWM and PM+ duration of sessions and adherence rates.

According to self-reports and structured observations, DWM and PM+ were delivered as intended. Implementer-reported duration of sessions revealed that most DWM and PM+ sessions lasted approximately 15 and 60 min, respectively. Structured observations performed by senior psychiatrists and clinical psychologists knowledgeable of the intervention programmes also revealed adherence rates of 90%–100% for DWM and 72%–90% for PM+. An overview of the results is shown in Figure 2.

#### Acceptability

The attrition rate for the complete stepped-care programme was 35%. Non-use attrition was low: two people did not access the DWM website (2/55 = 4%), and six did not attend any PM+ sessions (6/36 = 17%). Overall, DWM participants preferred synchronous support (i.e., 15-minute weekly phone calls) over asynchronous contacts (i.e., weekly messages within the DWM website), with only two participants choosing the latter after the DWM welcome call.

During phase 3, we used individual interviews with participants to explore three key intervention elements. We asked them their thoughts about having received support from psychiatry, clinical psychology and mental health nursing trainees, who were mostly under 30 and had no previous experience with this programme. One trial participant said that it was ‘exciting’ for residents and interns to ‘participate in these projects’ (TP5). Another participant mentioned familiarity:

So, let's see, I think that makes it more familiar. It feels closer. I appreciated it because I would have more reservations if I had come across someone my dad's age, I don't know. I think I was able to let go a little bit more just by seeing a young face, you know? (TP3)

Importantly, one participant did not see that this familiarity or closeness could affect confidentiality: ‘I’m the opposite. They give me more, and I feel like sharing. It's like they would understand me better, and I would be more comfortable’ (TP6).

Even though we did not ask decision-makers specifically about acceptability, one mentioned that intervention providers should be closely supervised:

All types of psychological distress, even if they're work-related, have their subtleties and complications. Indeed, dealing with second-year residents without supervision would not be appropriate, but having a sound supervision system seems to be an excellent way to approach it and learn. (DM3)

And added:

However, a person who starts the intervention and has the opportunity to test it will see that the good thing about residents is that they put in an effort and take the cases seriously. It may be essential to clarify that an expert will provide supervision. (DM3)

We also asked participants about remote delivery of DWM and PM+, which was seen as a key enabling factor linked to flexibility by both intervention providers and decision-makers. One participant said that if it had been delivered in person, many people would have probably not enrolled:

Unless there is an exercise that needs to be done face-to-face, it seems to me that the online format is good. You don't waste time getting to the consultation and other things that could put many people off because we don't have the time; we lack time now on all sides. (TP7)

Similarly, another participant said that people do not need to spend time to go to the sessions, providing flexibility:

I believe face-to-face is complicated and not as flexible for someone to get to one place and do it in person. I'm not sure what you have in mind for upcoming phases, but I think doing it online gives you much freedom. (TP3)

Lastly, we explored the overall acceptability and satisfaction with the stepped-care format. We could not recruit any participants who dropped out, that is, participants who did not complete the intervention. One participant who did not step up to PM+ due to a significant improvement in psychological distress said that that the level of support received ‘was enough’: ‘I had stress because something very specific was going on. After some months it stopped, and I started feeling better’ (TP1).

Participants who stepped up to PM+ also mentioned that it could be more feasible and even appropriate.

Then, I understand that if it's stepped, it's because there are a series of filters, and if a person meets a score, that is when they really continue. I believe it is okay, and in my case, I'm glad I did it because it worked. (TP3)

### Mechanisms of action

During phase 3, we asked trial participants if the interventions worked for them – and, if so, how. Participants often reported that the stepped-care programme worked because (a) they trust its effectiveness and this helped them to maintain adherence to the intervention:

And secondly, the fact that this was going to help me in some way, well, I had to do my part and find time to do it. And also, naturally, seeing that I was making progress little by little, well, it motivated me to continue. (TP3)

And (b) because of the good relationship with intervention providers:

He could empathise and listen, letting you know he's there, that he listens and understands you; in other words, it was mind-blowing. Maybe I had the extraordinary luck of relying on someone who helped me only by talking to him, that is, only by sharing and seeing how he listened to me. Look, I might even get a little bit emotional because I wasn't doing well, and he helped me a lot, so if you see me emotional, it's nothing but gratitude. (TP4)

In addition, they highlighted some specific components, especially breathing, grounding and mindfulness exercises, problem-solving techniques and tips for detaching or unhooking from difficult or unhelpful thoughts and feelings. For instance, a trial participant mentioned how they tried to put these techniques into practice: ‘Yes, the truth is that it’s made me think about many things, to be more focused, and as I have activities there, well, I try to put them into practice – the breathing itself’ (TP6) and another one mentioned that ‘above all, the breathing exercises, so being mindful at all times, in other words, grounding [were useful]’ (TP9). Other participants mentioned the following:

Mindfulness was like focusing on what I was doing at the moment. I took the example of having a cup of tea in the mornings or having a coffee, and not taking it in a rush, but being conscious of what I was doing and enjoying and savouring that it is hot and the cup. (TP2)

It helped me a lot, but I needed more, and I remember to put into practice, now that I'm back in the office, things that I talked about with your colleague, especially the management table of solvable and non-solvable problems and splitting them into little pieces. Then, with that and what I discussed with him, I decided what to do when I went back to practice after finishing the sessions. (TP8)

We did not record any serious adverse events during the RCT (phase 2), and during phase 3, we did not ask for unexpected pathways specifically.

## Discussion

We conducted a process evaluation of the RESPOND-HCWs trial, a RCT of a stepped-care programme for healthcare workers (HCWs) with psychological distress. We used the eHealth adaptations of Doing What Matters in Times of Stress (DWM) and Problem Management Plus (PM+), which were delivered fully remotely by psychiatry, clinical psychology and mental health nursing trainees. Overall, the process evaluation results complement the outcome evaluation of the trial, showing that the stepped-care programme was not only effective in reducing psychological distress^19^ but also feasible, appropriate, delivered in compliance with the manuals and well-accepted by the participants. To inform future implementation efforts, we identified key barriers (e.g., misleading expectations, mental health-related stigma) and enabling factors (e.g., schedule flexibility, remote delivery). We also flagged mechanisms of action that included common factors and intervention-specific ingredients, which may guide future content and delivery adaptations and research designs.

### Implementation outcomes: How was the stepped-care programme delivered?

Three indicators suggested that the RCT was feasible: the minimal delays and good completion rates in the collection of the primary outcome, the large number of participants who showed interest in being screened to participate in the trial in a short period of time and the involvement of intervention providers and supervisors employed by the Madrilenian Department of Health. The initial engagement of users can be explained by the accessible screening procedures (completely remote, minimal waiting periods) and the relatively short assessments (10–15 minutes each), both described in the study protocol.^12^ After being enrolled, participants may have completed most assessments due to the automatic reminders (for a competing view, see Sarathy et al.^27^) and real-time peer support – which may serve as an additional reminder for participants in the intervention arm.^28^ Furthermore, we involved end-users in designing and evaluating the trial, which can improve recruitment in mental health studies.^29,30^ Further trials may involve the public in the design, conduct, and dissemination of eHealth studies, not only to potentially increase feasibility, but also to foster social practices of dialogue and shared knowledge.^31^

The programme was also seen as appropriate, useful and timely, both by trial participants and decision-makers. When the study started in December 2020, our main concern was that HCWs may have already lost interest in receiving mental health support by the time recruitment started because COVID-19 could be seen as already in the past. The local adaptation process in early 2021 quickly reduced such concern: local stakeholders systematically underlined persistent mental health problems and unmet needs among HCWs in the Madrilenian Department of Health,^16^ results that were rapidly confirmed by ongoing cohort studies in Spain.^32–34^ Consequently, we argue that this intervention was not only appropriate, useful or timely but also necessary in the aftermath of the COVID-19 pandemic, considering the lack of evidence-based interventions to support HCWs in epidemics and other crisis settings.^20,21^ Notably, decision-makers also reported that the intervention was compatible with the organisational culture of the Madrilenian Department of Health NHS (as one respondent stated ‘We should look after the caregivers’). This is in line with the numerous supportive strategies deployed by the Spanish National Health System to support frontline workers during the pandemic^35^ and may potentially help improve implementation outcomes overall.^36,37^

In addition to feasibility and appropriateness, our process evaluation shows that both DWM and PM+ were delivered in compliance with the intervention protocols – adherence rates were slightly lower for PM+, probably due to the increased duration and complexity of the sessions. This is the first time DWM has been used as guided self-help outside Self Help Plus (SH+); hence, direct comparison is not feasible. However, DWM is similar to another WHO-guided self-help intervention called step-by-step, which has been tested in three RCTs so far. Overall, these trials showed minimal protocol deviations^38,39^ and good adherence to the duration of the 15-minutes weekly calls,^40^ in line with our results. Regarding PM+, the body of research is more extensive (for a recent review, see Schäfer et al.^41^) and fidelity checklists are not always included. Trials including external supervision of sessions reported adherence rates that were satisfactory overall, but hard to compare due to different checklist scoring.^42–44^ Only two studies defined adherence as the externally reported percentage of protocol tasks covered during the session, as we did, and they found adherence rates roughly similar to ours.^45,46^ Fidelity assessments are often overlooked in eHealth implementation studies of psychological interventions^47^ notwithstanding their implications for research. On the one hand, fidelity checks supplement outcome evaluations by increasing our confidence in the fact that the estimated effects are brought by the intervention itself, as defined in the intervention manual and not by other confounding factors. On the other hand, they help identify common protocol deviations that may emerge when programmes are scaled up – for example sessions longer than planned or pieces of content systematically skipped. In RESPOND-HCW, we aimed to offer an evidence-based intervention that could be delivered by healthcare staff in training at a large scale in crisis settings.

The last implementation outcome was acceptability, as measured by participants’ attrition rates and self-reports obtained through in-depth interviews performed after the trial. Overall, only one in three participants discontinued treatment, which aligns with average attrition estimates in RCTs of Internet-delivered interventions.^48,49^ Importantly, non-use attrition rates were minimal for DWM and low for PM+ in contrast to the 40% rate in the Step-by-Step trial in Lebanon^39^ and the overall low acceptance of guided self-help interventions for depression.^50^ This finding may be due to the local adaptation and tailoring process involving end-users and other stakeholders, which aligns with recommendations for eHealth and mHealth studies^51–53^ and the weekly support calls. Notably, even though this study focuses on the Madrilenian trial, where the only official language is Spanish, the intervention was delivered either in Spanish or Catalan in Catalonia, which might have made users feel more comfortable with it. Regarding PM+, non-use attrition is hard to interpret because it was offered as part of stepped care for the first time. Although we could not interview participants who were offered a step-up but decided not to attend any PM+ session, it is plausible that they dropped out as they felt that DWM was sufficient for them. Moreover, one interviewee who did not step up to PM+ due to significant clinical improvements after DWM said that the first step had been enough for her. On the other hand, PM+ requires more time, and it may not be feasible for some participants to spend one hour on weekly sessions. Nonetheless, our study fails to provide detailed data on the acceptability of the stepped-care format because people who dropped out rejected participating in the interviews. Future studies should explore these hypotheses to understand attrition drivers in different implementation contexts.

In addition to the stepped-care format, we explored whether people found it acceptable to use an intervention that was delivered fully remotely and provided by peers who did not have previous experience delivering the programme. Interviewees were happy with the remote format overall, although we found reports acknowledging the benefits of in-person support. This contrasts with a recent umbrella review, which reported reluctance towards mHealth interventions, such as DWM, during the COVID-19 pandemic.^6^ We argue that our stepped-care programme was well accepted because it was flexible and peer-delivered. Flexibility made our schedules more compatible with working shifts and caring duties, which are still performed mainly by women in Spain.^54^ This is particularly important because both HCWs and users of psychological interventions are mostly women (see below a discussion of flexibility as an enabling factor). Further, unlike most eHealth and mHealth interventions, DWM and, in particular, PM+ included individualised peer support. Considering that guided eHealth interventions are more effective than unguided ones,^55–58^ it is plausible that they are also more acceptable and satisfying. However, this result needs to be taken cautiously because people who dropped out from the intervention were underrepresented in our sample – and so were probably users who did not find the programme so acceptable and did not feel comfortable sharing this opinion with the research team. Nevertheless, a qualitative study that explored reasons for discontinuing usage of an internet-delivered intervention targeted at HCWs also identified the lack of support as an important barrier to the programme's use and convenient access as an enabling factor.^59^

Regarding intervention providers’ profiles, trial participants did not report any issues with them being young or not very experienced. This contrasts with the qualitative evaluation of the Step-by-Step trial in Lebanon, where most participants who dropped out would have preferred experts over peers.^60^ Even considering contextual influences such as the social, economic, and cultural environment, we cannot rule out the possibility that some RESPOND-HCWs participants discontinued treatment due to this. However, the more experienced profile of our helpers, compared to other settings, as expected, reduced the chances of such a perception. Of note, we did find some concern among decision-makers regarding intervention providers’ profiles – particularly mental health nursing trainees. Although this does not inform acceptability, it reveals a potential reluctance among implementers during scaling-up and should be carefully considered along with other potential barriers.

### Barriers, enabling factors, and mechanisms of action: how can the programme be improved?

A key barrier to participation frequently reported by trial participants and decision-makers was having unrealistic expectations about the intervention, as some expected intensive psychotherapy instead of peer-delivered, low-intensity stepped-care. In Spain, HCWs were exposed to high levels of stress for an extended time, during the initial outbreak and prolonged pandemic period,^32–34,61–63^ and half of the participants in our RCT reported baseline symptoms compatible with major depressive disorder.^19^ Even though we excluded people with severe mental problems, it was plausible that a low-intensity mHealth course like DWM was perceived as insufficient by some participants. The final decisions on designing and delivering a stepped-care programme depend on the needs and resources of specific contexts and settings. To support HCWs with psychological distress working in a pandemic hotspot, interventions had to be delivered rapidly and widely by non-expert providers. This hampered the deployment of more intense interventions (e.g., 12-session individual CBT), but it could have included an additional short orientation session to help participants adjust their expectations. Further studies could explore whether an explanatory video or a short introductory call from a research assistant or intervention provider significantly increases requirements, considering that it can help with additional barriers identified in this study and in previous trials with similar interventions,^60,64^ namely technical difficulties, tips for scheduling weekly practice or outstanding questions about confidentiality.

An additional barrier reported by participants and decision-makers was mental health-related stigma. This finding has been reported in previous process evaluations of step-by-step^60^ and PM^+^,^64^ and it is of great importance because stigma about mental health is associated with lower levels of mental health help-seeking behaviours worldwide.^65,66^ Interestingly, HCWs’ attitudes have been central in many mental-health-related stigma studies, but more as (de)stigmatisation agents than recipients.^67^ However, HCWs can suffer from stigma and discrimination as much as any other person with mental health problems. For example, in the United Kingdom, HCWs with a history of depression sought less mental health support than those without a history of depression – and these help-seeking behaviours were linked to perceived mental-health-related stigma.^68^ In Spain, amidst the COVID-19 pandemic, we found that only one in four HCWs with probable major depressive disorders in 2020 received psychological support in 2021 and 2022,^69^ which, in light of the present study, may also be partially explained by perceived stigma. To fight this persistent barrier, implementation strategies should benefit from a key enabling factor reported by most decision-makers in our study: the increasing sensitivity towards mental health problems within the Spanish healthcare system. This opportunity aligns with the global framework for action on mental health developed by the WHO, which aims to ‘mainstream, promote and safeguard mental well-being as an integral element of COVID-19 response and recovery’ and ‘counter the stigma and discrimination associated with mental health conditions’,^70^ as well as with the first mental health legislation proposal in Spain.^71^ In this context, there is a need to implement comprehensive mental health strategies that include anti-stigma campaigns specifically targeting healthcare and other essential workers who were distinctively affected by the COVID-19 pandemic.

Trial participants and decision-makers also identified another key enabling factor: the flexibility of schedules and delivery formats. Interventions like SH+ (which includes the DWM booklet) or PM+ were designed to be easily scaled-up in crisis settings, especially in conflict zones (e.g.,^44,72–74^). To our knowledge, this is the first study to offer these interventions fully remotely and with such flexible schedules. This was crucial to fit potential users’ changing schedules and the demands of lockdowns and social restrictions and could critically enhance scalability in similar crisis settings. Depending on the setting and available resources, a tradeoff between intervention providers’ availability and large-scale delivery should be carefully considered when designing implementation strategies.

To better understand DWM and PM+ mechanisms of action, we asked participants what had worked for them. According to the contextual model, psychotherapy produces benefits not only through specific ingredients of distinct psychotherapies but also through non-specific (i.e., common) factors, namely expectations and user-provider relationships.^75^ Participants identified specific ingredients that helped them, such as mindful breathing in DWM or problem-solving in PM+, but the most frequently reported factors were the confidence in the intervention's positive outcomes (i.e., expectations) and getting along very well with intervention providers (i.e., user–provider relationships). This indirect finding cannot solve the outstanding debate of whether psychotherapy works through common factors, specific ingredients, or a combination of both^76^ once again, it underscores the importance of personal guidance regardless of the specific intervention being offered. It is also compatible with findings of a trial that compared two formats of stepped-delivery of CBT against treatment as usual, where both active interventions were equally effective, somewhat suggesting that changes were determined by common factors or therapists’ characteristics.^77^

## Limitations

We identified some limitations that warrant a cautious interpretation of our results. First, we did not have independent raters to assess intervention providers’ protocol adherence. Second, we did not systematically register intervention providers’ attendance at supervision sessions. This was unnecessary because all absences were justified, either by annual/sick leave or through work duties, but this limits the possibility of external checks. Third, we did not use validated measures of satisfaction with DWM and PM+, which would have complemented qualitative data on acceptability and helped influence decision-making. Fourth, we failed to recruit people who dropped out of the trial for the process evaluation. This could lead to a non-response bias (i.e., a type of sampling bias that happens when participants do not provide data or drop out of the study) and affect the validity and transferability of our results. On the one hand, non-respondents could possess different characteristics and distinct motivations. For instance, they might not care as much about the topic or have a less positive experience with the intervention. Their lived experience would have been important in getting a better impression of key implementation outcomes, including acceptability or feasibility. On the other hand, participants who voluntarily decided to participate may show stronger or positive opinions about the intervention, positively skewing the results. Moreover, there was a lack of dropout rates among interviewees, which could indicate that they were engaged with the study. Fifth, the process evaluation was carried out in one trial location only. The Community of Madrid and Catalonia have substantial societal and political differences, including the structure of their Departments of Health. Process evaluations are useful if they are linked to local contexts, especially if mixed methods are used, and this would have exceeded the purpose of this analysis. Sixth, the qualitative analysis of the in-depth interviews with trial participants and decision-makers could benefit from a higher degree of inter-coder reliability. Although discrepancies were discussed with a second coder, most transcripts relied on a single researcher. Having multiple coders could strengthen the findings, increasing the robustness of the analysis by mitigating the risks associated with individual bias or misinterpretation. Seventh, we did not systematically assess thematic saturation. Some themes were consistently reported by many interviewees while others were reported less frequently, suggesting that a larger sample could bring at least some additional information to the analysis. Last, we focused on early implementation outcomes. Further studies should use strong designs and analyse late implementation outcomes, such as sustainability and penetration (i.e., the integration of the programme within the service setting), as well as other core indicators, particularly uptake and reach.

## Implications

Our results have important implications for research and practice. In terms of research, we call for trials to spend time locally adapting and tailoring the intervention and analysing the implementation context and outcomes – as we did, respectively, in phases 1 and 3. This may prevent unexpected consequences, including negative results (e.g., no intervention effects) and bad process outcomes (e.g., underpowered analyses due to high attrition).

In terms of practice and policy, our results support the implementation of remotely delivered stepped-care programmes for HCWs in crisis settings. Furthermore, our findings suggest that unspecific factors, such as the expectations or the therapeutic alliance, play a key role and that specific ingredients could be adapted or modified across different settings. Although our results cannot be simply transferred to other contexts, the currently tested WHO interventions DWM and PM+ were explicitly designed to be easily scaled up in different crisis settings, as they are contextually adaptable and can target different populations affected by adversity. For instance, additional process evaluations of ongoing RESPOND trials are being conducted. These studies target different population groups, such as refugees and asylum seekers in Italy, people without stable working conditions in France, and migrant workers in the Netherlands.^13–15^ These studies will help detect commonalities across populations and settings with potential implications for implementation research.

To achieve good implementation outcomes, we make the following recommendations. First, the programme must be flexible to fit in HCWs’ schedules, increase adherence rates, and potentially maximise effectiveness. Second, peer support is essential and should be included in all steps. Third, occupational- and community-level actions may enhance HCWs’ help-seeking behaviours and reduce mental health-related stigma. Fourth, including a short orientation session may promote more plausible expectations about the relatively short, low-intensity interventions. Finally, considering the key role of unspecific factors such as the expectations or the therapeutic alliance, specific ingredients could be adapted or modified across contexts. Additional process evaluations of ongoing RESPOND trials^13–15^ involving different population groups will help detect commonalities across populations and settings with potential implications for implementation research.

## Conclusions

This eHealth stepped-care programme is effective and potentially scalable based on the results of this process evaluation. Moreover, considering previous studies and the fact that these interventions were designed to be widely applicable and easily adaptable, the eHealth adaptations could be used in other crisis settings, such as conflict zones and across different vulnerable populations. Public health policies and occupational health actions should involve key stakeholders, especially end-users, and adapt DWM and PM+ to specific contexts to improve engagement. Policymakers should take advantage of their flexibility during scaling up without limiting access to more intensive support, including in-person psychological interventions.

## Supplemental Material

sj-docx-1-dhj-10.1177_20552076241287678 - Supplemental material for Beyond effectiveness in eHealth trials: Process evaluation of a stepped-care programme to support healthcare workers with psychological distress (RESPOND-HCWs) Supplemental material, sj-docx-1-dhj-10.1177_20552076241287678 for Beyond effectiveness in eHealth trials: Process evaluation of a stepped-care programme to support healthcare workers with psychological distress (RESPOND-HCWs) by Roberto Mediavilla, Blanca García-Vázquez and Kerry R. McGreevy, James Underhill, Carmen Bayón, María-Fe Bravo-Ortiz, Ainoa Muñoz-Sanjosé, Josep Maria Haro, Anna Monistrol-Mula, Pablo Nicaise, Papoula Petri-Romão, David McDaid, A-La Park, Maria Melchior, Cécile Vuillermoz, Giulia Turrini, Beatrice Compri, Marianna Purgato, Rinske Roos, Anke B. Witteveen, Marit Sijbrandij, Richard A. Bryant, Daniela Fuhr, José Luis Ayuso-Mateos in DIGITAL HEALTH

sj-docx-3-dhj-10.1177_20552076241287678 - Supplemental material for Beyond effectiveness in eHealth trials: Process evaluation of a stepped-care programme to support healthcare workers with psychological distress (RESPOND-HCWs) Supplemental material, sj-docx-3-dhj-10.1177_20552076241287678 for Beyond effectiveness in eHealth trials: Process evaluation of a stepped-care programme to support healthcare workers with psychological distress (RESPOND-HCWs) by Roberto Mediavilla, Blanca García-Vázquez and Kerry R. McGreevy, James Underhill, Carmen Bayón, María-Fe Bravo-Ortiz, Ainoa Muñoz-Sanjosé, Josep Maria Haro, Anna Monistrol-Mula, Pablo Nicaise, Papoula Petri-Romão, David McDaid, A-La Park, Maria Melchior, Cécile Vuillermoz, Giulia Turrini, Beatrice Compri, Marianna Purgato, Rinske Roos, Anke B. Witteveen, Marit Sijbrandij, Richard A. Bryant, Daniela Fuhr, José Luis Ayuso-Mateos in DIGITAL HEALTH
