# Supplementary material for: Beyond effectiveness in eHealth trials: Process evaluation of a stepped-care programme to support healthcare workers with psychological distress (RESPOND-HCWs)
Source: Digit Health. 2024 Oct 18;10:20552076241287678. doi: 10.1177/20552076241287678 (PMC11489946; doi:10.1177/20552076241287678)

**Beyond Effectiveness in eHealth Trials: Process Evaluation of a Stepped-care Programme to Support Healthcare Workers with Psychological Distress in Crisis Settings (RESPOND-HCWs)**

**Supplemental Material**

[Supplementary Table 1. *Characteristics of the participants in Phase 1* 2](#_Toc176256999)

[Supplementary Table 2. *Characteristics of the participants in Phase 2* 3](#_Toc176257000)

[**Supplementary File 1. Interview Script** 4](#_Toc176257002)

[*Interview script: trial participants 4*](#_Toc176257003)

[*Interview script: decision-makers 7*](#_Toc176257004)

[*Focus group script: intervention providers 9*](#_Toc176257005)

[**Supplementary File 2. Supervision Checklist** 14](#_Toc176257006)

[Supervision Checklist: *Doing What Matters in Times of Stress (DWM)* 14](#_Toc176257007)

[Individual Problem Management Plus (PM+) Helper’s Component Checklist 15](#_Toc176257008)

[Supplementary Figure 1. *DWM and PM+ components* 20](#_Toc176257009)

# **Supplementary Table 1.** *Characteristics of the participants in Phase 1*

#### Free list (FL) interviews Key informant (KI) interviews

|  | **Total** | **Madrid** | **Catalonia** |  | **Total** | **Madrid** | **Catalonia** |
| --- | --- | --- | --- | --- | --- | --- | --- |
|  | **(n** = **75)** | **(n** = **41)** | **(n** = **34)** |  | **(n** = **22)** | **(n** = **10)** | **(n** = **12)** |
| **Age group** |  |  |  |  |  |  |  |
| 18–35 | 23 | 14 | 9 |  | 7 | 3 | 4 |
| 36–50 | 42 | 17 | 15 |  | 14 | 6 | 8 |
| >50 | 20 | 10 | 10 |  | 1 | 1 | 0 |
| **Gender** |  |  |  |  |  |  |  |
| Female | 46 | 21 | 25 |  | 14 | 6 | 8 |
| Male | 29 | 20 | 9 |  | 8 | 4 | 4 |
| **Job** |  |  |  |  |  |  |  |
| Frontline worker | 37 | 17 | 20 |  | 13 | 5 | 8 |
| Mental health expert | 26 | 16 | 10 |  | 8 | 4 | 4 |
| Administrators and service planners | 12 | 8 | 4 |  |  |  |  |
| **Facility** |  |  |  |  |  |  |  |
| Hospital | 36 | 17 | 19 |  |  |  |  |
| Non-hospital | 27 | 16 | 11 |  |  |  |  |

Note. Adapted from Mediavilla R. (16).

# **Supplementary Table 2.** *Characteristics of the participants in Phase 2*

|  | | **Group** | |
| --- | --- | --- | --- |
|  | **Overall, N = 232** | **Control, N = 117** | **Intervention, N = 115** |
| **Age, M (SD)** | 37.5 (10.3) | 37.1 (10.4) | 37.9 (10.1) |
| **Gender, n (%)** |  |  |  |
| Female | 200 (86%) | 99 (85%) | 101 (88%) |
| Male | 32 (14%) | 18 (15%) | 14 (12%) |
| **Educational level** |  |  |  |
| Secondary | 1 (0.4%) | 0 (0%) | 1 (0.9%) |
| Technical-professional | 41 (18%) | 18 (15%) | 23 (20%) |
| University | 190 (82%) | 99 (85%) | 91 (79%) |
| **Type of job** |  |  |  |
| Physician | 50 (22%) | 28 (24%) | 22 (19%) |
| Nurse | 130 (56%) | 66 (57%) | 64 (56%) |
| Nursing technician | 29 (13%) | 12 (10%) | 17 (15%) |
| Administration | 6 (2.6%) | 1 (0.9%) | 5 (4.3%) |
| Other | 16 (6.9%) | 9 (7.8%) | 7 (6.1%) |
| **Job facility** |  |  |  |
| Hospital facilities | 147 (63%) | 72 (62%) | 75 (65%) |
| Primary care facilities | 68 (29%) | 35 (30%) | 33 (29%) |
| Specialised care facilities | 5 (2.2%) | 3 (2.6%) | 2 (1.7%) |
| Emergencies | 10 (4.3%) | 6 (5.1%) | 4 (3.5%) |
| Other | 2 (0.9%) | 1 (0.9%) | 1 (0.9%) |
| **Frontline worker (ever)** | 215 (93%) | 108 (92%) | 107 (93%) |
| **COVID-19 infection (ever)** | 137 (59%) | 69 (59%) | 68 (60%) |
| **Site** |  |  |  |
| Madrid | 110 (47%) | 55 (47%) | 55 (48%) |
| Catalonia | 122 (53%) | 62 (53%) | 60 (52%) |

## Note. Adapted from Mediavilla R. (27). M = mean, SD = standard deviation.

## **Supplementary File 1. Interview Script**

## **Interview script:** *trial participants*

Introduction

- Greet the person. Introduce yourself
- Remember the participant that he/she agreed to being called after the trial for an in-depth interview when he/she signed the informed consent form (you can forward it to the participant if required). Right after that, briefly present the study: “We would like to ask you some questions about your experience of this program to help us to think about how it could be improved for delivery in the future. There are no right or wrong answers to the questions we are going to ask. We will be speaking to a number of people that participated in the project, asking everyone the same questions. If you feel unable to answer a question, please say so and we will move on to the next one.”
- “Do you agree to participate? We will be recording the session for transcription purposes. We will erase the tape as soon as we transcribed it. The transcription will be stored securely and will not have any personal data linked to it”

Overall impressions

- Could you tell me a little bit about yourself? (E.g., where do you work, what do you fancy, and so on) [Informal, brief first contact]
- Could you tell me how you found out about the RESPOND stepped-care programme (remember it is a two-step intervention and we offered PM+ after step one if the person was still distressed)?
- How have you found using this support programme?
  - What did you like about it?
  - What didn’t you like or think could be improved?
  - Was it like you expected it to be?
- Did you share with family and/or friends about your involvement in the programme? [keep it very short, especially if the person does not engage]
  - Explore reasons for sharing/not sharing
  - If shared with family*:* Could you tell more about what you shared about the programme with your family/friends/others?
  - Explore whether just shared about their participation in the programme or also details about the skills learned during the intervention and their experiences of the intervention
  - How did your family view your participation in the PM+ programme?

Mechanisms

- What did you find useful about the intervention?
- What did you find less useful about the intervention?
- Overall, would you say the intervention worked for you?
  - Why? How?

Implementation

- How did you find the online format?
  - Explore DWM: app, ongoing support calls, audios, journal, etc.

*Only for participants that continued to PM+:*

- - Explore PM+: videocalls, length, schedules
- How did you find the stepped-care format?
  - How did you feel about (not) being offered PM+?
- Can you describe how you found working with your helper?
  - Explore positive / negative views through probes
  - Explore acceptability of features of facilitators, i.e. HCWs in training, sometimes from the same organization, often young, online format
- Did you feel that you were being supported by an expert or by a peer/colleague?

Context

- Can you describe how easy or difficult you found attending DWM? *(if applicable)* And what about PM+?
  - Explore barriers and facilitators to attendance (e.g. timing in the week; *for DWM:* focus on the weekly support calls, , *for PM+:* flexibility of timing of sessions, time commitment needed for attending sessions and doing homework, combining with childcare/household responsibilities/paid work)
  - Explore if the role of helpers and anything they were doing / saying that encouraged attendance.

*Only for participants that continued to PM+:*

- Can you identify any factors that motivated you to attend each week / what was it that encouraged you to come back to the session each week?
  - Explore i.e. community views of the program; seeing improvements made them want to come back; family encouragement etc.
  - Explore if the role of helpers and anything they were doing / saying that encouraged attendance.
- Can you describe how you found implementing the strategies taught to you in your everyday routine?
  - Explore DWM strategies (INCLUDE)
  - Explore PM strategies (INCLUDE)
- Did you continue with the skills you learned as part of the programme after the last session?
  - Explore reasons for (not) doing so
- [FOR DROP-OUTS ONLY] Can you tell me why you stopped going through the DWM app / attending the PM+ sessions?
  - Explore reasons through probes (i.e., not having an impact, difficulties in relationship with helper, unable to commit to N weekly sessions, length or location of the session, other commitments – family/work, etc.)
  - IF APPROPRIATE: Explore possible ways to overcome barriers (e.g., do you think one would be better able to complete the programme if there are less than 5 sessions?)
- What do you think are the main reasons why a HCWs would not choose to participate in a programme like this? [time commitment, stigma, e-help, helper’s characteristics, programme content]
- In your view, do you think there is a need for an intervention like this for HCWs in Madrid/Catalonia? Please explain [advanges over existing mental health services, beneficial only for some, need for different forms of the programme, i.e., face-to-face, group, etc.]

Wrap-up

- Ask the interviewee if they have anything to add. Any additional information is added to the interview notes as required.
- Thank person and leave.

## **Interview script:** *decision-makers*

Introduction

- Greet the person. Introduce yourself
- We sent you a brief that introduces the RESPOND project, which we have been carrying out in Madrid and in Catalonia. As you know, we are exploring the effectiveness of a peer-delivered, online-based psychological intervention. In this interview, we are interested in understanding more in depth how we can scale up this intervention within the Madrilenian health system, for which we would like to hear your opinion. The interview will last 20-30 minutes.
- “Do you agree to participate? We will be recording the session for transcription purposes. We will erase the tape as soon as we transcribe it. The transcription will be stored securely and will not have any personal data linked to it”.

Overall impression

- What do you think about the intervention programme?
  - What is your opinion about transferring it from settings without mental health providers into the SERMAS?
  - Was it timely?
- What do you think about the stepped-care format?

Context

- In your view, do you think there is a need for an intervention like the stepped-care (DWM and PM+) RESPOND to be provided on a larger scale in Spain?
  - Explore possible advantages over existing mental health services and the need for achieving higher coverage of treatment for psychological problems
- We are interested to know more about scaling up of RESPOND stepped-care program. By scaling up we mean making it accessible to all HCWs in Madrid/Catalonia by integrating it into the health system. Based on what you’ve just heard/learned about RESPOND, where in the system in Spain could you see the intervention being provided? Please explain [multiple options possible].
  - In which settings within the SERMAS/DSGC?
  - To which specific groups (e.g., nurses, PC doctors, etc.)
  - Who do you think should provide it?
  - Could you think of something similar? If yes, can it be integrated in there?
  - Explore how can it be funded in the long-term (e.g. health insurance, (local) government, private or donor funds)
- What do you think are the main obstacles for scaling up RESPOND in Madrid/Catalonia [refer to possible pathways/systems discussed in previous question; and discuss barriers for each]?
  - Explore possible obstacles through probes like the ones below. Not all probes need to be covered; better to select probes based on background/expertise of person being interviewed and those not yet covered in interviews with similar interviewees.
    - Structure (‘ways of organising’): e.g. how to ensure sufficient and sustainable human and financial resources to scale up the programme; who could provide it (someone within SERMAS/DSGC or outside SERMAS/DSGC); how to ensure physical access for all potential users (e.g. time/costs acceptable, any differences rural/urban and men/women); is there sufficient political will; how to upkeep effectiveness of the intervention during implementation
    - Practice (‘ways of doing’): e.g. how can the intervention be made part of existing care and referral pathways (e.g. stepped-care); how can quality and safety of RESPOND be ensured (e.g. continued training and supervision of helpers); how to prevent RESPOND being a burden (emotional/time/financial) to helpers and supervisors
    - Culture (‘ways of thinking’): e.g. how can we ensure stigma surrounding mental health and help seeking will not be a major obstacle during scale up; how can we ensure that non-professional or less-trained providers will be accepted by other health professionals; is there sufficient need/demand for and how can we best reach the target group(s)
- Thank you for explaining some of the obstacles. I am interested to hear your thoughts on what is needed to overcome these obstacles (facilitators); could you tell me more about this?
  - Explore ways to overcome the obstacles mentioned in previous question
- What are your views of having lay counselors as providers?
  - Explore positive and negative views through probes
  - Explore whether having lay counsellors as service providers is something new and if not ask for examples of other initiatives
- How do you think quality control and accountability can be best be ensured when scaling-up [name intervention]?
  - Explore what can be done when there is spontaneous scaling-up/mushrooming and how then to ensure quality and safety of the intervention
- Finally, do you know of any wider trends that have happened or are current happening in Madrid, which could positively or negatively influence the scaling up of PM+?
  - Ask about possible political developments, refugee integration policies (work, education), economic developments, socio-cultural climate (acceptance of refugees)
  - Ask about the possible influence of pandemics like COVID-19

Wrap-up

- Ask the interviewee if they have anything to add. Any additional information is added to the interview notes as required.
- Thank person and leave.

## **Focus group script:** *intervention providers*

| **FGD Guide helpers for wp4** | |
| --- | --- |
| **Objective** | To explore helpers’ experience in the RESPOND project and their thoughts on implementing the DWM/PM+ programme in *[name country]*. |
| **Research question** | What is the experience of helpers on the RESPOND project and how can this help accommodate implementation of the stepped-care DWM/PM+ program? |
| **Sub-questions** | - What is helpers’ general experience of the RESPOND project?   - How did helpers experience the DWM programme?   - How did helpers experience the PM+ programme? - What challenges did helpers experience while working as a helper?   - Challenges during DWM?   - Challenges during PM+? - What challenges did helpers see for participants in the RESPOND project?   - Challenges during DWM?   - Challenges during PM+? - What challenges do helpers see as the main challenges? - What do helpers think is needed in order to overcome the identified main challenges and implement the DWM/PM+ programme in *[name country]*? |
| **Target population** | Helpers in the RESPOND project |
| **Duration** | 1.5h |
| **Materials** | Post-its, stickers, flipcharts, pens, colored markers, papers for writing exercise (prepare them so people can just fill it in) |
| **Room preparation** | - Put tables together and enough seats for everyone - Flipchart, should not be visible beforehand - Flipchart – write in middle: ‘challenges to DWM’ - Flipchart – write in middle: ‘Challenges to PM+’ |
| **1 min** | 1. **Introduction**  - Welcome - Thank participants for taking time - Explain why they are at this FGD – what it will look like.   *The reason you are here is because all of you were helpers in the RESPOND project. We are very interested in your experience as a helper, and your opinion on the DWM app and PM+, which we want to explore through different exercises.* |
| **4 min** | 1. **Practical issues**   Emphasize the following points   - *There are no right or wrong answers, so feel free to speak your mind.* - *There are no obligations during this discussion, but I will really appreciate your active participation* - *This is a confidential discussion and material gathered will only be shared among the research team members* - *The FGD will take about 1.5 hours* - *We will have a break midway* - *Questions?* |
| **5 min** | 1. **Introductory exercise** (just to get into the right mindset)   Exercise 1  *For the past [x time] you have all been working as a helper. [give short summary they can relate to]. As a start, I want you to think back of everything you have done these past [x months].*   - Discuss with neighbour, 5 minutes - State that this exercise is only to remember everything they have done and to get ready for the following exercises. |
| **10 min** | 1. **Overall impression**   Exercise 2  *Now that you all have had a quick chat about your time as a helper on the project, I want to get to know more about your experience. Please go to mentimeter. Write all key words that come to mind when you think of working as a helper on the RESPOND project.*   - State that now they all have had a quick chat about their time as a helper on the project, you want to get to know more about their experience. - Ask participants to think of their experience as a helper, and write all key words that come to mind in sticky notes - **General discussion** on key words – cluster them with other words and place them in a big paper   - Probe where needed for clarification, but don’t go in too deep   - Try to get a general sense of what it was like for them to be a helper |
| **30 min** | 1. **DWM** |
|  | Exercise 3   - Explain that we want to explore if and how we can best implement the DWM app in *[name country]*. - Important to have a clear idea of challenges they envision when using DWM in practice   *When you think of the DWM app, what challenges did you experience? Think of challenges for helpers as well as challenges for participants. Write each challenge on a post-it.*   - Give participants 5 minutes - After 5 minutes, ask if everyone is ready. If everyone seems to be done sooner, then ask this sooner - Put a flipchart in the middle of the table on which is written: ‘Challenges to DWM’ - Ask if anyone wants to **share what they wrote**. - Probe to understand their perspective: - What do you mean? - What makes you feel that way? - How come you wrote this?  etc 🡪 make sure it’s clear if it’s a challenge for helpers or for participants. - Ask participant to **put post-it on flipchart** with ‘challenges to DWM’ written on it. - Ask if someone wrote something similar. If yes, put next to post-it that it’s most similar to, so you naturally create groups of similar challenges next to each other. - Try to have a group discussion on it - **Cluster** the challenges together with participants:  - state that you want to cluster the challenges - point to a group of post-its, ask if they think it can be seen as a cluster. - if yes: of what? If no: what is the cluster? How would they group them? - draw a circle around each cluster of challenges, and name them. - **Summarize** what challenges have been stated when using DWM in practice and what clusters have been formed, e.g. mainly challenges for helpers, or challenges for participants, practical issues etc., relationship with participants. - Ask if some challenges are missing / have a short group **discussion** on it. Note: you don’t want to give suggestions about topics for challenges as to not bias people, but do explore until you’re certain that all challenges related to implementing DWM have been covered.   *Possible answers – challenges can be related to:*   - *Using the app for participants* - *Using the app as a helper* - *Completing the app* - *Weekly support calls* - *Relationship with participants* - *Peer delivery* - *Etc.* |
|  | *** BREAK *** |
|  | 1. **PM+** |
|  | Exercise 4 (same as exercise 3)   - Explain that we want to explore if and how we can best implement the PM+ in *[name country]*. - Important to have a clear idea of challenges they envision when using PM+ in practice   *When you think of PM+, what challenges did you experience? Think of challenges for helpers as well as challenges for participants. Write each challenge on a post-it.*   - Give participants 5 minutes - After 5 minutes, ask if everyone is ready. If everyone seems to be done sooner, then ask this sooner - Put a flipchart in the middle of the table on which is written: ‘Challenges to PM+’ - Ask if anyone wants to **share what they wrote**. - Probe to understand their perspective: - What do you mean? - What makes you feel that way? - How come you wrote this?  etc 🡪 make sure it’s clear if it’s a challenge for helpers or for participants. - Ask participant to **put post-it on flipchart** with ‘challenges to PM+ written on it. - Ask if someone wrote something similar. If yes, put next to post-it that it’s most similar to, so you naturally create groups of similar challenges next to each other. - Try to have a group discussion on it - **Cluster** the challenges together with participants:  - state that you want to cluster the challenges - point to a group of post-its, ask if they think it can be seen as a cluster. - if yes: of what? If no: what is the cluster? How would they group them? - draw a circle around each cluster of challenges, and name them. - **Summarize** what challenges have been stated when delivering PM+ in practice and what clusters have been formed, e.g. mainly challenges for helpers, or challenges for participants, practical issues etc., relationship with participants. - Ask if some challenges are missing / have a short group **discussion** on it. Note: you don’t want to give suggestions about topics for challenges as to not bias people, but do explore until you’re certain that all challenges related to implementing PM+ have been covered.   *Possible answers – challenges can be related to:*   - *Scheduling sessions* - *Delivering sessions – which in particular?* - *Engagement of participants* - *Relationship with participants* - *Peer delivery* - *Etc.* |
| **5 min** | **DWM + PM+** |
|  | Exercise 5   - Discuss common challenges between DWM and PM+ - Double check if anything is missing   What do you think of the RESPOND stepped-care programme within *[NAME COUNTRY]*?  What are the main obstacles for providing the intervention for more people in *[name country]*?   - Make sure you have all challenges covered - Ask participants to reach consensus on how to order them -from most to least important |
| **15 min** | **Implementation** |
|  | Exercise 6 - writing exercise   - For the identified main challenges in exercise 5, chose the challenges with the most votes, equal to the number of helpers. - Each participant takes one of the papers and writes one of the identified main problems on the paper - Then they write a proposed solution (1 min) - All papers go to the next participant to the right of them, and they write a problem they see with the proposed solution, and suggest how this can be overcome (emphasize that it’s ok if they don’t know something) (1 min) - Do this until each participant has had the opportunity to write a solution for each challenge   Writing exercise:  *Writing exercise:*   1. *Problem:* 2. *How can this be solved?* 3. *What problem do you see with this solution?* 4. *How can this be overcome?* 5. *What problem do you see with this solution?* 6. *How can this be overcome?*  - Discuss the barriers and solutions one by one. Whoever wrote last presents it. - As a group, discuss the outcome |
| **2 min** | **Ending**   - State that these were the exercises - Thank participants for their contribution - Ask participants what they learned in this FGD? - Ask how they felt about the FGD: what did they like? What did they like less? - Any questions/remarks? |

## **Supplementary File 2. Supervision Checklist**

## **Supervision Checklist:** *Doing What Matters in Times of Stress (DWM)*

CALL ID: _____________ (EXAMPLE: rmt_seg5_abc) SUPERVISOR ID: __________ (AMS, EFJ, JCR, ILR)

| Step | Checklist | Date *(if completed)* | Details |
| --- | --- | --- | --- |
| Pre-Service Planning | | | |
| 1. Reading and reviewing the manual for guided self-help | ☐ Completed  ☐ Not completed | N/A | Leave blank if does not apply or cannot be inferred |
| 1. Reading the stress management guide/online intervention and practicing the skills | ☐ Completed  ☐ Not completed | N/A | Leave blank if does not apply or cannot be inferred |
| Welcome call | | | |
| 1. Introducing the stress management online intervention | ☐ Completed  ☐ Not completed | N/A |  |
| 1. Describe the helper’s role (not completed if helper describes role as exceeding that of a helper e.g. offering therapy etc) | ☐ Completed  ☐ Not completed | N/A |  |
| 1. Discuss confidentiality | ☐ Completed  ☐ Not completed | N/A |  |
| 1. Discuss the stress management online intervention (not completed if spends majority of call discussing topics beyond the scope of the intervention and does not bring discussion back to focus on the intervention) | ☐ Completed  ☐ Not completed | N/A |  |
| 1. Schedule a follow up | ☐ Completed  ☐ Not completed | N/A |  |
| Ongoing calls | | | |
| 1. Greeting | ☐ Completed  ☐ Not completed | N/A |  |
| 1. Review user’s experience of the module | ☐ Completed  ☐ Not completed | N/A |  |
| 1. Discuss practicing the exercises/techniques | ☐ Completed  ☐ Not completed | N/A |  |
| 1. Schedule next meeting | ☐ Completed  ☐ Not completed | N/A |  |
| 1. Helper provides support within limit of role (e.g. discusses topics beyond the scope of the intervention and does not bring discussion back to focus on the intervention) | ☐ Completed  ☐ Not completed | N/A |  |

## **Individual Problem Management Plus (PM+) Helper’s Component Checklist**

This form is a session-by-session checklist helpers can use when delivering the Individual PM+ program. The main components can be marked off or circled when they have been completed for each session.

SESSION 1

| No. | Item | Components Checklist  (Circle or mark all that apply/that are completed) |
| --- | --- | --- |
| 1.1 | Conduct **Introductions** and explain  **Confidentiality** | 1.1a – Introduce yourself  1.1b – Explain concept of confidentiality, including information about when confidentiality can be broken  1.1c – Answer participant questions about PM+ intervention and sessions if needed  1.1.d – Informed consent for audio recordings |
| 1.2 | Introduce **What is PM+?** | 1.2a – Explain PM+ intervention to the participant  1.2b – Discuss participant’s reasons for and challenges to attending PM+ sessions  1.2c – Support participant to manage any obstacles to attending sessions |
| 1.3 | Conduct **What is Adversity** | 1.3a – Define adversity using participant’s examples  1.3b – Discuss and normalise common reactions to adversity  1.3c – Discuss how PM+ aims to help participants manage their problems |
| 1.4 | Teach and Practice **Managing Stress Exercise** | 1.4a – Provide information on how stress affects the body  1.4 b – Relate the information to participant’s physical/tension problems  1.4 c – Teach and practice breathing from the diaphragm/stomach (show balloon)  1.4 d – Practice slow breathing together  1.4 e – Discuss challenges and difficulties |
| 1.5 | Uses appropriate **psychosocial communication skills** | 1.5a – Appropriate eye contact, facial expression, and body language  1.5b – Demonstrates a non-judgmental attitude  1.5c – Appropriate use of non-verbal communication  1.5d – Communicates concern and validates participant |
| 1.6 | Incorporates **safety management skills** | 1.6a –Reviews for suicidality if necessary  1.6 b - Identifies potentials risks of harm to self or others  1.6c – Uses techniques for acute management of risk and provides referral |
| 1.7 | **Closing Procedures** | 1.7a - Review of session and schedule home practice  1.7b- Information of next session (remind date, time, place and strategy) |

SESSION 2

| No. | Item | Components (Circle or mark all that apply) |
| --- | --- | --- |
| 2.1 | Incorporates **safety management skills** | 2.1a –Reviews for suicidality if necessary  2.1 b - Identifies potentials risks of harm to self or others  2.1c – Uses techniques for acute management of risk and provides referral |
| 2.2 | Welcome and review **Managing Stress** | 2.2a – Welcomes the participant back  2.2b – Discuss questions participant has about Session 1  2.2c – Review participant’s Managing stress home practice  2.2d – Help manage any difficulties with home practice |
| 2.3 | Introduce **Managing Problems** | 2.3a – Introduce strategy Managing problems for practical problems  2.3b – Explain each of the 7 steps  2.3c – Help the participant to apply the strategy to a chosen problem  2.3d – Help the participant develop and action plan  2.3e – Give participant Managing Problems handout |
| 2.4 | Practice **Managing Stress** | 2.4a – Practice slow breathing together  2.4b – Discuss challenges and difficulties |
| 2.5 | Uses appropriate **psychosocial communication skills** | 2.5a – Appropriate eye contact, facial expression, and body language  2.5b – Demonstrates a non-judgmental attitude  2.5c – Appropriate use of non-verbal communication  2.5d – Communicates concern and validates participant |
| 2.6 | Incorporates **safety management skills** | 2.6a –Reviews for suicidality if necessary  2.6b - Identifies potentials risks of harm to self or others  2.6c – Uses techniques for acute management of risk and provides referral |
| 2.7 | **Closing Procedures** | 2.7a - Review of session and schedule home practice  2.7b- Information of next session (remind date, time, place and strategy) |

SESSION 3

| No. | Item | Components (circle or mark all that apply) |
| --- | --- | --- |
| 3.1 | Incorporates **safety management skills** | 3.1a –Reviews for suicidality if necessary  3.1 b - Identifies potentials risks of harm to self or others  3.1c – Uses techniques for acute management of risk and provides referral |
| 3.2 | Welcome and review **Managing Stress** | 3.2a – Welcomes the participant back  3.2b – Discuss questions participant has about previous sessions  3.2c – Review participant’s Managing stress home practice  3.2d – Help manage any difficulties with home practice |
| 3.3 | Review **Managing Problems** | 3.3a –Discuss participant’s experiences of completing their Action Plan for Managing Problems  3.3b – Respond to and manage any difficulties (e.g. unable to complete, encountered problems when completing)  3.3c – Help participant apply strategy to continue managing the same problem or a new problem |
| 3.4 | Introduce **Get Going and Keep Doing** and the Inactivity cycle | 3.4a – Introduce Get Going and Keep Doing strategy  3.4b – Show the Inactivity Cycle and explain  3.4c – Discuss how inactivity cycle can be broken  3.4d – Give participant Get Going and Keep Doing handout |
| 3.5 | Apply **Get Going Keep Doing** with an enjoyable activity | 3.5a – Help participant to select an **enjoyable** activity  3.5b – Help participant break down their activity into small steps  3.5c - Help participant develop their action plans |
| 3.6 | Practice **Managing Stress** | 3.6a – Practice slow breathing together  3.6b – Discuss challenges and difficulties |
| 3.7 | Uses appropriate **psychosocial communication skills** | 3.7a – Appropriate eye contact, facial expression, and body language  3.7b – Demonstrates a non-judgmental attitude  3.7c – Appropriate use of non-verbal communication  3.7d – Communicates concern and validates participant |
| 3.8 | Incorporates **safety management skills** | 3.8a –Reviews for suicidality if necessary  3.8b - Identifies potentials risks of harm to self or others  3.8c – Uses techniques for acute management of risk and provides referral |
| 3.9 | **Closing Procedures** | 3.9a - Review of session and schedule home practice  3.9b- Information of next session (remind date, time, place and strategy) |

SESSION 4

| No. | Item | Components (circle or mark all that apply) |
| --- | --- | --- |
| 4.1 | Incorporates **safety management skills** | 4.1a –Reviews for suicidality if necessary  4.1 b - Identifies potentials risks of harm to self or others  4.1c – Uses techniques for acute management of risk and provides referral |
| 4.2 | Welcome and review **Managing Stress** | 4.2a – Welcomes the participant back  4.2b – Discuss questions participant has about previous sessions  4.2c – Review participant’s Managing stress home practice  4.2d – Help manage any difficulties with home practice |
| 4.3 | Review **Managing Problems** | 4.3a –Discuss participant’s experiences of completing their Action Plan for Managing Problems  4.3b – Respond to and manage any difficulties (e.g. unable to complete, encountered problems when completing)  4.3c – Help participant apply strategy to continue managing the same problem or a new problem |
| 4.4 | Review **Get Going Keep Doing** | 4.4a – Discuss participant’s experiences of completing their Action Plan for Get Going Keep Doing  4.3b – Respond to and manage any difficulties (e.g. unable to complete, encountered problems when completing)  4.3c – Help participant apply strategy to continue with the same activity or start a new activity (task-oriented activity) |
| 4.5 | Introduce **Strengthening** **Social Support** | 4.5a – Define Strengthening Social Support  4.5b – Discuss different social supports and how they can be helpful  4.5c – Give participant Strengthening Social Supports handout |
| 4.6 | Apply **Strengthening Social Support** | 4.6a – Help participant identify how they can strengthen their social support  4.6b – Help participants develop an action plan  4.6c – Rehearse with participants what they will do/say in their action plan if applicable |
| 4.7 | Practice **Managing Stress** | 4.7a – Practice slow breathing together  4.7b – Discuss challenges and difficulties |
| 4.8 | Uses appropriate **psychosocial communication skills** | 4.8a – Appropriate eye contact, facial expression, and body language  4.8b – Demonstrates a non-judgmental attitude  4.8c – Appropriate use of non-verbal communication  4.8d – Communicates concern and validates participant |
| 4.9 | Incorporates **safety management skills** | 4.9a –Reviews for suicidality if necessary  4.9b - Identifies potentials risks of harm to self or others  4.9c – Uses techniques for acute management of risk and provides referral |
| 4.10 | **Closing Procedures** | 4.10a - Review of session and schedule home practice  4.10b- Information of next session (remind date, time, place and strategy) |

SESSION 5

| No. | Item | Components (circle or mark all that apply) |
| --- | --- | --- |
| 5.1 | Incorporates **safety management skills** | 5.1a –Reviews for suicidality if necessary  5.1 b - Identifies potentials risks of harm to self or others  5.1c – Uses techniques for acute management of risk and provides referral |
| 5.2 | Welcome and review **all PM+ home practice** | 5.2a – Welcomes the participant back  5.2b – Discuss questions participant has about previous sessions  5.2c - Review Managing Stress home practice  5.2d – Review Managing Problems home practice  5.2e – Review Get Going Keep Doing home practice  5.2f – Review Strengthening Social Support home practice  5.2g – Manage any difficulties participants had with any of their home practice |
| 5.3 | Complete **Staying Well** | 5.3a – Congratulate the participant on finishing PM+  5.3b – Discuss participant improvements and areas for continued work  5.3c – Emphasise importance of continuing to practice PM+ strategies, using learning a new language example  5.3d – Discuss potential future problems and how participant can respond |
| 5.4 | Complete **How to help others** | 5.4a – Use the case examples to discuss how the participant could help others experiencing problems  5.4b – Discuss their responses and correct any misunderstandings |
| 5.5 | Complete **Looking Forward** | 5.5a – Review goals that were not achieved  5.5b – Discuss how the participant can work towards these goals  5.5b – Help participants to identify new goals |
| 5.6 | Uses appropriate **psychosocial communication skills** | 5.6a – Appropriate eye contact, facial expression, and body language  5.6b – Demonstrates a non-judgmental attitude  5.6c – Appropriate use of non-verbal communication  5.6d – Communicates concern and validates participant |
| 5.7 | Incorporates **safety management skills** | 5.7a –Reviews for suicidality if necessary  5.7b - Identifies potentials risks of harm to self or others  5.7c – Uses techniques for acute management of risk and provides referral |
| 5.8 | **Closing Procedures** | 5.8a – Congratulate the participant and wish them the best for the future  5.8b – Remind them they will be contacted to complete the post-assessment |

# **Supplementary Figure 1.** *DWM and PM+ components*


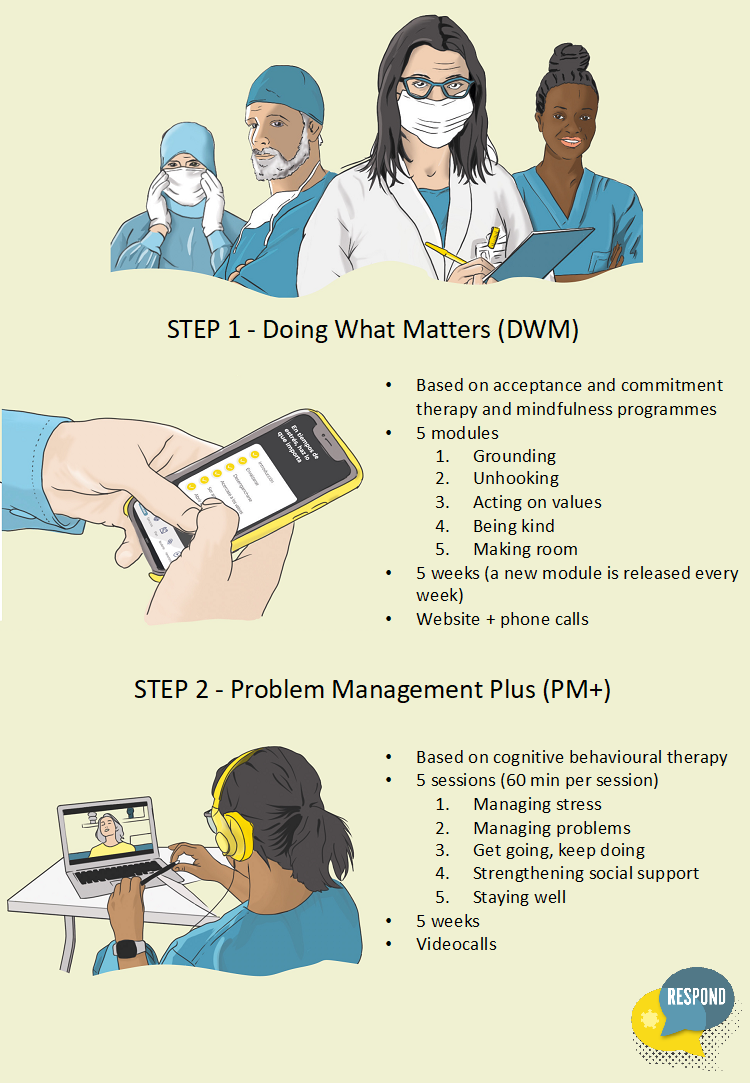

Supplement: sj-docx-1-dhj-10.1177_20552076241287678 - Supplemental material for Beyond effectiveness in eHealth trials: Process evaluation of a stepped-care programme to support healthcare workers with psychological distress (RESPOND-HCWs) [file sj-docx-1-dhj-10.1177_20552076241287678.docx]
